# Promising novel therapy with hydrogen gas for emergency and critical care medicine

**DOI:** 10.1002/ams2.320

**Published:** 2017-10-24

**Authors:** Motoaki Sano, Masaru Suzuki, Koichiro Homma, Kei Hayashida, Tomoyoshi Tamura, Tadashi Matsuoka, Yoshinori Katsumata, Shuko Onuki, Junichi Sasaki

**Affiliations:** ^1^ Department of Cardiology Department of Emergency and Critical Care Medicine Keio University School of Medicine Tokyo Japan

**Keywords:** Acute kidney injury, critical care, hydrogen, myocardial infarction, out‐of‐hospital cardiac arrest

## Abstract

It has been reported that hydrogen gas exerts a therapeutic effect in a wide range of disease conditions, from acute illness such as ischemia–reperfusion injury, shock, and damage healing to chronic illness such as metabolic syndrome, rheumatoid arthritis, and neurodegenerative diseases. Antioxidant and anti‐inflammatory properties of hydrogen gas have been proposed, but the molecular target of hydrogen gas has not been identified. We established the Center for Molecular Hydrogen Medicine to promote non‐clinical and clinical research on the medical use of hydrogen gas through industry–university collaboration and to obtain regulatory approval of hydrogen gas and hydrogen medical devices (http://www.karc.keio.ac.jp/center/center-55.html). Studies undertaken by the Center have suggested possible therapeutic effects of hydrogen gas in relation to various aspects of emergency and critical care medicine, including acute myocardial infarction, cardiopulmonary arrest syndrome, contrast‐induced acute kidney injury, and hemorrhagic shock.

## Introduction

The efficacy of molecular hydrogen (hydrogen gas) for the prevention and treatment of various diseases has been reported by numerous non‐clinical and clinical studies, and the multiple effects of hydrogen are attracting attention.

Research on molecular hydrogen has primarily been initiated in Japan, achieving impressive results without notable adverse reactions. We established the Center for Molecular Hydrogen Medicine to promote non‐clinical and clinical research on the medical use of hydrogen gas through industry–university collaboration and to obtain regulatory approval of hydrogen gas and hydrogen medical devices (http://www.karc.keio.ac.jp/center/center-55.html). Studies undertaken by the Center have suggested possible therapeutic effects of hydrogen gas in relation to various aspects of emergency and critical care medicine, and have played a strategic role in pioneering research on medical uses of hydrogen.

Up to 12 L hydrogen gas is produced daily by the intestinal flora, but the molecular mechanisms underlying the effects of hydrogen gas at very low concentrations (1–4%) have not been fully elucidated. Initially, attention was focused on its ability to eliminate reactive oxygen species. Therefore, a number of experiments were carried out to verify the preventive effect of hydrogen gas on ischemia–reperfusion injury. We also started with animal and clinical studies to verify the therapeutic effect of hydrogen gas on myocardial ischemia–reperfusion injury. Excessive production of reactive oxygen species (ROS), mainly in the mitochondria, plays a key role in cellular damage associated with ischemia–reperfusion injury, but elimination of ROS by conventional antioxidants is less than effective. This apparent paradox is explained by the dual nature of ROS. Destructive ROS like hydroxyl radicals are strong oxidants that cause tissue damage, whereas beneficial species like superoxide and hydrogen peroxide enhance endogenous antioxidant mechanisms through signal transduction pathways. A potent antioxidant, such as vitamin C, indiscriminately eliminates both destructive and beneficial ROS, thus failing to suppress the onset or progression of conditions related to oxidative stress. Hydrogen gas is a weak reducing agent, and its oxidation‐reduction reaction only occurs with a strong oxidant that causes tissue damage.^1^ Because of its low molecular weight, hydrogen can readily diffuse through cell membranes to rapidly reach the mitochondria where ROS are generated or the nucleus where genetic information is stored, and can protect these organelles from oxidative injury. However, as research progresses, many biological effects of hydrogen gas have been documented that cannot be explained simply by the removal of ROS based on its own reducing effect. For example, in cardiopulmonary arrest syndrome, although ROS are thought to be the most strongly involved in pathology at the time when return of spontaneous circulation (ROSC) is achieved, the improvement of prognosis of cerebral function by hydrogen gas inhalation therapy is achieved even if hydrogen gas administration is started after a certain time after ROSC. Furthermore, hydrogen gas inhalation confers resistance to hemodynamic instability caused by massive bleeding. Although it is a vague idea, it appears that, when hydrogen gas is administered under circumstances where homeostasis is disturbed, it works on complex networks and restores homeostasis. Endogenous physiologically active gases, such as nitric oxide and carbon monoxide, bind to heme, but hydrogen gas does not. There is a difficulty in finding the target molecules of hydrogen gas here. At the present time, the mechanism of action is unknown, and only the versatile therapeutic effects are confirmed.

In this review article, we will introduce the basic and clinical research related to emergency and critical care medicine that we have undertaken so far.

## Properties of hydrogen gas

Among various methods for administration of hydrogen gas, we selected inhalation because it allows monitoring of the dose of hydrogen. Hydrogen gas is flammable and is combustible over the range of 4.0–75.0 vol% (hereinafter described as %) in air at room temperature (also known as the explosion limit). The explosion limit of hydrogen in oxygen ranges between 4.0 and 94.0%. Hydrogen gas is less likely to self‐ignite, with the ignition point being higher for it (527°C) than for gasoline (500°C). Hydrogen gas at concentrations of less than 4.0% used for basic and clinical research is not flammable, and there is no risk of combustion or explosion. When hydrogen is released into the air, it undergoes dispersal and there is no risk of combustion provided that the original concentration of gaseous hydrogen is under the explosion limit. Hydrogen has the lowest molecular weight of all molecules and hydrogen gas promptly becomes undetectable due to diffusion in air or even through walls because of its low density.

## Clinical evidence

### Acute myocardial infarction

The most effective therapy for ST elevation acute myocardial infarction (MI) is rapid reperfusion of the culprit vessel to minimize the infarct size. Paradoxically, reperfusion itself enlarges the infarct size due to reperfusion injury. Thus, the final infarct size after MI is determined by the combination of ischemic myocardial damage and secondary myocardial injury associated with reperfusion (i.e., ischemia–reperfusion injury).

Efforts to reduce the infarct size are critical to prevent harmful left ventricular remodeling and/or development of heart failure. As cardiomyocytes cannot regenerate, heart transplantation or cell transplantation (regenerative medicine) are the only therapies that can replace lost cells after MI. However, the infarct size might be minimized if reperfusion is carried out at the appropriate timing and ischemia–reperfusion injury is suppressed.

The blood and tissue levels of hydrogen reach saturation within 2 or 3 min after commencing inhalation of hydrogen gas. The gaseous hydrogen level in the blood reaches 16 μmol/L after inhalation of 2% hydrogen gas. Arterial oxygen saturation is not affected because gaseous hydrogen does not bind with hemoglobin, and the blood pressure and pulse rate are also unaffected under steady state condition. The blood level of hydrogen gas declines rapidly after discontinuation of inhalation because it is excreted by the lungs.^2^

We undertook experiments in rats^2^ and dogs^3^ with Professor Shigeo Ohta from the Nippon Medical School (Tokyo, Japan) and Dr. Masafumi Kitakaze from the National Cerebral and Cardiovascular Center (Osaka, Japan), which verified that inhalation of 1–4% hydrogen gas alleviated tissue damage and reduced the infarct size. An investigator‐initiated clinical trial to assess the safety and efficacy of inhaled hydrogen gas for the prevention of reperfusion injury in patients with acute MI undergoing percutaneous coronary intervention was started at Keio University Hospital (Tokyo, Japan) in December 2011 (UMIN Clinical Trials Registry number: UMIN000006825). High‐pressure gas cylinders were filled with a mixture of hydrogen (1.3%), oxygen, and nitrogen that was directly inhaled by the patients. It was found that inhalation of hydrogen gas did not reduce the infarct size during the acute phase of MI. However, comparison of cardiac magnetic resonance imaging data obtained 1 week and 6 months after MI showed no change or a decrease in left ventricular stroke volume in the control group, whereas it was increased in the hydrogen gas inhalation group.^4^ This suggested that inhalation of hydrogen gas during the acute phase of MI suppressed adverse left ventricular remodeling at 6 months after infarction.

### Out‐of‐hospital cardiac arrest

The Ministry of Internal Affairs and Communications and the Fire and Disaster Management Agency of Japan have reported that out‐of‐hospital cardiac arrest affects 100,000 persons annually, including 60,000 with cardiogenic cardiac arrest. Among them, 20,000 arrests are witnessed. Even these 20,000 patients with the most favorable prognosis among all those with out‐of‐hospital cardiac arrest have a very low survival rate of 8%, and only 4% achieve social rehabilitation. While survival has improved somewhat with wider knowledge of cardiopulmonary resuscitation and use of hypothermia, serious sequelae stemming from brain damage are common even if resuscitation is successful, and the patient has a very slim chance of returning to work. The very serious state that develops after spontaneous circulation is restored following cardiac arrest is generally called the post‐cardiac arrest syndrome (PCAS), and new methods other than hypothermia for protecting the brain from this syndrome are needed to improve the chances of achieving social rehabilitation.

In 2012, we established a rat model of resuscitated cardiac arrest, in which cardiopulmonary resuscitation was carried out by chest compression and ventilation with 98% oxygen following arrest for 5 min due to ventricular fibrillation (VF) induced by delivery of high‐frequency electric current to the chest wall. After cardiopulmonary resuscitation, the rats were divided into a control group with a target temperature of 37°C, a hypothermia group with a target temperature of 33°C, a hydrogen gas group with a target temperature of 37°C plus inhalation of 2% hydrogen gas, and a combined hypothermia/hydrogen group with a target temperature of 33°C plus inhalation of 2% hydrogen gas. The survival rate at 72 h after ROSC was only 30% in the control group, whereas it increased to 70% in the hypothermia group and the hydrogen gas group, and was even higher at 80% in the combined group. The neurological outcome (evaluated by a neurological deficit score) at 72 h after resumption of circulation was better in the hypothermia group and in the hydrogen gas group compared with the control group.^5^

Nevertheless, this preliminary experiment revealed some problems to be solved. First, inhalation of hydrogen gas improved the neurological prognosis in terms of the neurological deficit score at 24 h after ROSC, but the cerebroprotective effect of inhaling hydrogen gas also needed verification at the histopathological level. Given that delayed neuronal death typically develops days or even weeks after ROSC, experimental conditions were required that would allow survival of the test animals for 7 days or longer after resuscitation. Second, both inhalation of hydrogen gas and hypothermia were started at the time of cardiopulmonary resuscitation, but a patient with ROSC is only likely to receive hydrogen inhalation and/or hypothermia after admission to an intensive care unit. It was therefore necessary to examine whether inhalation of hydrogen gas was still effective at some time after ROSC or was only effective if done at the time of ROSC, when production of ROS causing ischemia–reperfusion injury is elevated. Third, inhalation of oxygen at high concentrations does more harm than good because oxygen toxicity leads to cellular and tissue injury by ROS, but mixing hydrogen with inhaled oxygen can prevent such injury. Accordingly, the improvement seen when inhalation of hydrogen gas was combined with 98% oxygen might have merely represented neutralization of the harmful effect of such a high oxygen concentration.

Therefore, we modified the experimental protocol for resuscitation to solve these problems. In rats, the duration of cardiac arrest due to VF induced by high‐frequency electric current was prolonged to 6 min, and 100% oxygen inhalation was limited to 5 min after ROSC, followed by inhalation of 26% oxygen. Rats were divided into a control group, (target temperature, 37°C), a hypothermia group (target temperature, 33°C), a 2% hydrogen gas inhalation group, and a combined therapy group. Interestingly, the prognosis was markedly improved by limiting inhalation of a high oxygen concentration to 5 min after ROSC followed by inhalation at a lower concentration, even though the duration of cardiac arrest was prolonged by 1 min. It was possible to perform histopathological examination to compare the neurological outcome at 7 days after ROSC. A harmful effect of prolonged inhalation of oxygen at a high concentration was confirmed by this experiment. The survival rate at 7 days after ROSC was 38.4% in the control group, 71.4% in both the hypothermia group and the hydrogen gas group, and 85.7% in the combined therapy group. In the control group, histopathological examination at 7 days after ROSC showed notable neuronal degeneration and activation of microglia in the hippocampus, but these changes were suppressed in the hypothermia group and the hydrogen gas group, with even more marked suppression in the combined group. These results indicated that inhalation of hydrogen gas after ROSC was as effective as hypothermia for improving the neurological prognosis in rats with PCAS, while combined therapy had an additive effect.^6^

It is desirable to modify the oxygen level depending on the patient's condition, while maintaining a constant hydrogen gas level. When hydrogen is given with oxygen to a patient who has been resuscitated following out‐of‐hospital cardiac arrest, gaseous hydrogen is given through a ventilator. Because gases other than oxygen and air cannot be connected to a ventilator, hydrogen gas was added to the ventilator inhalation line, and a mixture of hydrogen and nitrogen was used to supply hydrogen gas safely. When a different gas is mixed into the inhalation line, the tidal volume indicated on the monitor of the ventilator differs from the volume shown on the patient's circuit. Similarly, the oxygen concentration will also differ from that set on the ventilator. Accordingly, a monitor was installed to accurately measure the ventilation volume and oxygen concentration delivered to the patient. After development of a device with these modifications, a clinical study of hydrogen gas inhalation was carried out in comatose patients with a consciousness level ≤8 points on the Glasgow Come Scale and a systolic blood pressure ≥90 mmHg (irrespective of vasopressor use) among the patients with out‐of‐hospital cardiopulmonary arrest transported to the Emergency Department of Keio University Hospital in 2015 (UMIN000012381). Of the five patients enrolled, one patient with cardiac arrest secondary to sepsis induced by pneumonia died 3 days after hospitalization. In this patient, the initial electrocardiogram showed pulseless electrical activity and total duration of cardiac arrest exceeded 40 min. The remaining four patients had cardiogenic cardiac arrest with VF on the initial electrocardiogram and the duration of arrest was approximately 20 min. All four patients were ambulatory at discharge from hospital with few neurological sequelae. Although many adverse events occurred, the independent data monitoring committee concluded that these events were consistent with the clinical course of PCAS and none were attributable to inhaling hydrogen gas. This study confirmed that inhalation of hydrogen gas could be performed safely in patients with out‐of‐hospital cardiac arrest.^7^ Subsequently, “inhalation of hydrogen gas in patients with PCAS” was approved as advanced medical care by the Ministry of Health, Labour and Welfare of Japan in November 2016. Then a multicenter study was started in February 2017 to verify the efficacy of hydrogen gas inhalation in patients with PCAS (http://www.hybrid2.org) (UMIN000019820).

## Areas where clinical application of hydrogen gas is expected in the near future

### Contrast‐induced acute kidney injury

Although contrast‐induced acute kidney injury (CIAKI) is usually reversible, blood purification is required or irreversible kidney dysfunction may develop in some patients and renal survival is shortened.^8^ Injection of contrast medium promotes secretion of endothelin, a vasoconstrictor peptide produce by endothelial cells, by the renal parenchyma and the resulting vasoconstriction reduces renal tissue blood flow and oxygen supply.^9^ Also, the osmotic pressure is elevated by infusion of contrast medium, which increases the amount of NaCl reaching the ascending limb of the loop of Henle, and increased sodium reabsorption accelerates renal oxygen consumption. This leads to renal medullary hypoxia and parenchymal injury due to accelerated production of free radicals by renal tubular epithelial cells.^10^ As contrast medium flows through the renal tubules, it gradually becomes highly concentrated, and toxicity of the agent itself can directly damage tubular epithelial cells.^11^, ^12^, ^13^ Emergency investigations and treatment often require use of a contrast medium, even in patients with a high risk of CIAKI. Infusion of fluid before and/or after use of contrast medium is currently recommended in patients with risk factors for CIAKI such as chronic kidney disease, old age, and diabetes. Drugs such as N‐acetylcysteine, human atrial natriuretic peptide (hANP), ascorbic acid, and statins were previously thought to be effective for the prevention of CIAKI, but none of them have been confirmed to be useful. The efficacy of blood purification for the prevention of CIAKI has also been denied by most reports. Prolonged infusion of fluid before administration of contrast medium is currently the only method recommended for the prevention of CIAKI, but it is not feasible in the emergency setting.

We created a rat model of CIAKI and used it to investigate the preventive effect of hydrogen gas inhalation.^14^ Rats inhaled hydrogen gas prior to infusion of contrast medium, and the renal function and histology were compared with rats that inhaled a control gas. It was found that hydrogen gas inhalation was effective in suppressing renal dysfunction induced by contrast medium. Immunostaining of renal tissues for 8‐OHdG, an index of oxidative stress, revealed significantly fewer 8‐OHdG‐positive cells in the hydrogen gas inhalation group, suggesting that suppression of oxidative stress by hydrogen gas was a factor in the prevention of renal dysfunction. As hydrogen gas was only inhaled during administration of the contrast agent, it is expected that this method could be effective for emergency patients requiring contrast imaging with no time for sufficient prehydration.

### Hemorrhagic shock

Hemostasis and transfusion are the standard countermeasures for hemorrhagic shock, but it is often impossible to perform transfusion promptly. For example, immediate blood transfusion is impossible if hemorrhagic shock develops due to injury at a remote location not readily accessible by ambulance, which is the case with many accidental injuries. Therefore, it is critical to stabilize the patient's condition and prolong survival until definitive treatment for hemorrhagic shock can be provided. Infusion of physiological saline to replace the lost blood is current emergency treatment for hemorrhagic shock, but fluid replacement is not always satisfactory in terms of prolonging survival. We recently reported that inhalation of hydrogen gas could improve and stabilize hemodynamics following fluid replacement in a rat model of hemorrhagic shock, and also dramatically improved survival after resuscitation.^15^

## Conclusion

Efficacy of molecular hydrogen for various diseases has been shown by basic research. When the clinical efficacy of hydrogen gas is confirmed and regulatory approval is obtained, the indications for this treatment will expand over time. In the near future, it is possible that hydrogen gas will be supplied to patients in the ambulance and will be available from standard wall outlets in hospital, as well as being provided for home inhalation after discharge. However, the clinical efficacy of hydrogen gas needs to be verified scientifically. Hydrogen gas has various physiological actions such as an antioxidant effect, anti‐inflammatory effect, and a protective effect against cell death, but the molecular mechanisms involved have not yet been clarified. The suppression of free radicals alone cannot explain the therapeutic effects of hydrogen. In the future, clarification of the target molecule should help to determine the optimum dosage of hydrogen gas and how to administer hydrogen gas for various indications. The particular advantage of using a cylinder to supply hydrogen gas is instant availability of highly pure gas at a stable concentration, but cylinders are disadvantageous for prolonged treatment. If hydrogen becomes widely used as a medical gas, a hydrogen generator that rapidly stabilizes the concentration and provides a sufficient flow rate would also need to be developed to allow prolonged inhalation of hydrogen gas.

## Disclosures

All animal experiments were approved by the Keio University Ethics Committee for Animal Experiments.

All clinical studies were approved by the Ethics Committee, Keio University School of Medicine and were registered in UMIN: The Safety and Efficacy of Inhalation of H2 Gas during PCI in Patients with Acute Myocardial Infarction (UMIN000006825); The Effect and Safety of Hydrogen Inhalation on Outcome Following Brain Ischemia during Post Cardiac Arrest Care: HYBRID study (pilot study) (UMIN000012381); and Hydrogen Inhalation Therapy for Patients with Post Cardiac Arrest Syndrome (phase II, multicenter, prospective, randomized, double‐blind, placebo‐controlled trial) (UMIN000019820).

Informed consent was obtained from all patients or their substitute for being included in the study.

Basic and clinical research was financed with the provision of funds from Taiyo Nippon Sanso.

Conflict of interest: None.

## References

[1] Ohsawa I , Ishikawa M , Takahashi K *et al* Hydrogen acts as a therapeutic antioxidant by selectively reducing cytotoxic oxygen radicals. Nat. Med. 2007; 13: 688–94. 17486089 10.1038/nm1577

[2] Hayashida K , Sano M , Ohsawa I *et al* Inhalation of hydrogen gas reduces infarct size in the rat model of myocardial ischemia‐reperfusion injury. Biochem. Biophys. Res. Commun. 2008; 373: 30–5. 18541148 10.1016/j.bbrc.2008.05.165

[3] Yoshida A , Asanuma H , Sasaki H *et al* H_2_ mediates cardioprotection via involvements of K(ATP) channels and permeability transition pores of mitochondria in dogs. Cardiovasc. Drugs Ther. 2012; 26: 217–26. 22527618 10.1007/s10557-012-6381-5

[4] Katsumata Y , Sano F , Abe T *et al* The effects of hydrogen gas inhalation on adverse left ventricular remodeling after percutaneous coronary intervention for ST‐elevated myocardial infarction ‐ first pilot study in humans. Circ. J. 2017; 81: 940–7. 28321000 10.1253/circj.CJ-17-0105

[5] Hayashida K , Sano M , Kamimura N *et al* H_2_ gas improves functional outcome after cardiac arrest to an extent comparable to therapeutic hypothermia in a rat model. J. Am. Heart Assoc. 2012; 1: e003459. 23316300 10.1161/JAHA.112.003459 PMC3541633

[6] Hayashida K , Sano M , Kamimura N *et al* Hydrogen inhalation during normoxic resuscitation improves neurological outcome in a rat model of cardiac arrest independently of targeted temperature management. Circulation 2014; 130: 2173–80. 25366995 10.1161/CIRCULATIONAHA.114.011848

[7] Tamura T , Hayashida K , Sano M *et al* Feasibility and safety of hydrogen gas inhalation for post‐cardiac arrest syndrome ‐ first‐in‐human pilot study. Circ. J. 2016; 80: 1870–3. 27334126 10.1253/circj.CJ-16-0127

[8] Stacul F , van der Molen AJ , Reimer P *et al* Contrast induced nephropathy: updated ESUR Contrast Media Safety Committee guidelines. Eur. Radiol. 2011; 21: 2527–41. 21866433 10.1007/s00330-011-2225-0

[9] Heyman SN , Brezis M , Epstein FH , Spokes K , Silva P , Rosen S . Early renal medullary hypoxic injury from radiocontrast and indomethacin. Kidney Int. 1991; 40: 632–42. 1745012 10.1038/ki.1991.255

[10] Bakris GL , Lass N , Gaber AO , Jones JD , Burnett JC Jr . Radiocontrast medium‐induced declines in renal function: a role for oxygen free radicals. Am. J. Physiol. 1990; 258: F115–20. 2301588 10.1152/ajprenal.1990.258.1.F115

[11] Itoh Y , Yano T , Sendo T *et al* Involvement of *de novo* ceramide synthesis in radiocontrast‐induced renal tubular cell injury. Kidney Int. 2006; 69: 288–97. 16408118 10.1038/sj.ki.5000057

[12] Schick CS , Haller C . Comparative cytotoxicity of ionic and non‐ionic radiocontrast agents on MDCK cell monolayers *in vitro* . Nephrol. Dial. Transplant. 1999; 14: 342–7. 10069186 10.1093/ndt/14.2.342

[13] Peer A , Averbukh Z , Berman S , Modai D , Averbukh M , Weissgarten J . Contrast media augmented apoptosis of cultured renal mesangial, tubular, epithelial, endothelial, and hepatic cells. Invest. Radiol. 2003; 38: 177–82. 12595799 10.1097/01.RLI.0000054529.61167.84

[14] Homma K , Yoshida T , Yamashita M , Hayashida K , Hayashi M , Hori S . Inhalation of hydrogen gas is beneficial for preventing contrast‐induced acute kidney injury in rats. Nephron. Exp. Nephrol. 2014; 128: 116–22. 10.1159/000369068 25592271

[15] Matsuoka T , Suzuki M , Sano M *et al* Hydrogen gas inhalation inhibits progression to the “irreversible” stage of shock after severe hemorrhage in rats. J. Trauma Acute Care Surg. 2017; 83: 469–75. 28640781 10.1097/TA.0000000000001620

